# Reincarnating the Identity Theory

**DOI:** 10.3389/fpsyg.2018.02044

**Published:** 2018-10-24

**Authors:** Erik Myin, Farid Zahnoun

**Affiliations:** Department of Philosophy, Centre for Philosophical Psychology, University of Antwerp, Antwerp, Belgium

**Keywords:** mind/body, identity theory, embodied cognition, multiple realization, experience

## Abstract

The mind/brain identity theory is often thought to be of historical interest only, as it has allegedly been swept away by functionalism. After clarifying why and how the notion of identity implies that there is no genuine problem of explaining how the mental derives from something else, we point out that the identity theory is not necessarily a mind/brain identity theory. In fact, we propose an updated form of identity theory, or embodied identity theory, in which the identities concern not experiences and brain phenomena, but experiences and organism-environment interactions. Such an embodied identity theory retains the main ontological insight of its parent theory, and by invoking organism-environment interactions, it has powerful resources to motivate why the relevant identities hold, without posing further unsolvable problems. We argue that the classical multiple realization argument against identity theory is built on not recognizing that the main claim of the identity theory concerns the relation between experience and descriptions of experience, instead of being about relations between different descriptions of experience and we show how an embodied identity theory provides an appropriate platform for making this argument. We emphasize that the embodied identity theory we propose is not ontologically reductive, and does not disregard experience.

## The Official Story

“The Identity Theory” forms, after treatments of dualism and behaviorism, the typical chapter 3 in an *Introduction to Philosophy of Mind* handbook. There, it will be narrated how Smart and Place, seeking to do justice to “inner” aspects of mind allegedly ignored by behaviorism, identified mental processes and states with brain processes and states, creating the mind/brain identity theory. Inevitably, the narrative will lead to the difficulties of the identity theory to deal with the phenomenal, the refutation of it by Putnam’s multiple realization arguments, and the subsequent replacement of the identity theory by functionalism.

Though this has, by all standards, become the official story, we think it should not be taken for granted. Quite the opposite. Contrary to this official story, some form of the identity theory – so we will contend – still offers the best available means to deal with the question of how minds and bodies relate. Moreover, contrary to reigning consensus, the identity theory has not been refuted by multiple realization arguments. However, an identity theory need not necessarily be a mind/brain identity theory. In particular, we think that it is possible to combine the idea of identity with an embodied or enactive view of the mind. Moreover, the result holds considerable explanatory potential. Or such we will defend in this paper.

## Identity and Explanation

The identity theory proposes to identify the mental with something else: somehow, what we call mental is not different from what we call physical or material. As we’ll see, it’s possible to develop different forms of identity theory from this root idea, theories, furthermore, which are standardly piled together and presented as the classic identity theory. Yet, so we think, it is the very idea of identity from which the main merits of these theories flow. The idea of strict identity, which lies at the heart of identity theories, is that something that we call by different names, or encounter in different ways, is despite initial appearances, actually not different, but identical, in the sense of being one and the same thing (see Smart, 1959). Identity theories, of whatever stripe, hold that this notion of strict identity forms the basis for an adequate response to the question of how the mental relates to the physical.

Consider one standard example used in discussions of the identity theory: the identification of the Evening Star with the Morning Star. The relevant identification consists in denying that, despite our initial impressions, there are two different entities at play here. If we thought that there were, we were wrong. Importantly, once the identification is made, the main explanatory task that we are left with is to understand why we previously missed the identity, or how it was possible for us to be misled by different appearances of the same object – different ways in which we encountered the single planet Venus. Of course, in coming to make the identification, we need to have reasons for making it. In the example, this will be that, on reflection, the different appearances show particular patterns in time and space. For example, when taking into account time differences due to location, we notice we can explain the timing of the appearances of the Evening Star and of the appearances of the Morning Star. Moreover, taking into account the different perspectives due to location, the appearances are of an object with the same shape and size. Crucially, while such facts motivate why it is reasonable to believe that the identity holds (see Hutto and Myin, 2013: 176), they don’t explain why Venus, under whatever description, has the properties that it has. For example, such facts can be referred to for justifying why someone holds that the Evening Star is the Morning Star, yet they don’t thereby explain why the Morning Star is identical to the Evening Star, or why the planet called Venus, Morning Star or Evening Star is as it is. In fact, identities such as the one that holds between the Evening Star and the Morning Star cannot be further explained^1^. They just hold, and one can either fail, or manage to be aware of them.

The reason why a strict identity cannot be explained, lies in the identity itself. For if there are explanations, for some X and Y (where these are real entities or events, not encounters with entities or events), of why X relates to Y, these typically are explanations in terms of how X causes, brings forth, or generates Y. But that is to say that X and Y are not identical to start with, for something can cause, bring forth, or generate only something which is different from itself. For an X and a Y which are identical, the idea of the one causing, bringing forth, or generating the other is non-sensical. To use another classic example, Clark Kent doesn’t cause Superman, he is Superman. It is possible to ask questions such as “Why is Clark Kent always where and when Superman shows up?” and the answer to such questions lies in pointing at the identity. Also, it is possible to ask “Why is Superman Clark Kent?” But the answer that one can provide to that question is one of explaining why we should believe in that identity, not one which offers some elucidating explanation of the identity itself, of why it holds, as distinct from why we should think that it holds.

If the usual example of the Morning Star and the Evening Star allows us to illustrate the, for our purposes, relevant aspects of what could be called the logic of identity, there’s something potentially misleading about it too. For there is nothing experiential or subjective (even in the minimal sense of essentially being tied to a person or subject) about that star (or rather: planet). The different ways of encountering it, which should not be mistaken for the encountering of different things, are of a different nature, being just different objective perspectives on an object. But with experience, this changes, because experience can be ‘encountered’ in different ways: it can be enacted, or embodied, by the subject of the experience, but it can also be encountered objectively, for example when it is observed by another subject.

Pursuing this difference requires that we first introduce a new species of identity theory.

## Embodied Identity Theory: Going Wide

The identity theory as proposed by Place, Feigl, and Smart did more than identifying the mental with the physical: it identified mental states and processes with brain states and brain processes.

Indeed, their identity theory was a mind/brain identity theory, and often these phrases are taken to be synonymous. Tellingly, the most outspoken recent defender of the identity theory, Thomas Polger, explicitly commits to a mind/brain identity theory (Polger, 2004).

But nothing in the idea of identity demands that the terms of identity be mind and brain, instead of mind and something else. As a consequence, it is possible to develop an identity theory in line with an embodied or enactive view of the mind (such E-views have been proposed by many, see Thompson, 2007; Barrett, 2011; Hutto and Myin, 2013, 2017). According to such views, experience and cognition are to be (re-) conceived in terms of organism-environment interactions. Sensation, perception, experience and cognition are “things organisms do,” and should be understood in terms of past and current interactions with the environment (Hutto and Myin, 2013). Explanations of experience, mind and cognition are subject to an “equal partner principle” (Hutto and Myin, 2013, p. 137) according to which environmental and intra-organismic factors can have equal weight in explanations of mental phenomena. The brain is seen as one of the players in the game, not as the locus of mindedness – that status is conferred to the spatially and temporally situated organism.

We here are not going to defend the legitimacy, or superiority of such embodied/enactive view of the mind an sich (though many, including the current authors have done so elsewhere, see Hutto and Myin, 2013, 2017, Zahnoun, 2018). Rather, our current goal is to show that one can combine an identity theory and the embodied/enactive view of the mind and to argue that such a combination of an embodied/enactive view of experience and an identity theory is not only possible, but eminently viable.

So what can be said in favor of an identity theory that “goes wide,” or holds that experience and other phenomena referred to as mental are identical to situated activities of organisms in environments? To start answering this, consider an identification in a murder case. What a murder is, constrains what, or rather who, the murderer can be identified with, namely, a human being. Given that a murder is a premeditated act in which one human being is killed by another human being, a murder case can only be laid to rest by identifying a previously unknown murderer with a human being. It would be out of the question to take an object or an animal as a possibility for identification. The specifics of a particular case further narrow down the possibilities for identification. Whoever is singled out as the murderer must have been at the right place at the right time, must have left traces, must have some plausible motivation or psychological history, and so on. The more it is shown that such constraints apply to an actual identification, the more belief in the identity is motivated (a phrase from Hutto and Myin, 2013, p. 175).

Now return to experience and the question of what it is to be identified with. The fact that a particular experience has the general characteristics that it has, such as being perspectival, subjective and affect-laden, exerts overall constraints on what it can be identified with. Activities of organisms fit the bill nicely, for they always have the required perspectivalness. They have a “value” uniquely related to a particular organism’s needs.

In addition, specific aspects of particular organism-environment interactions fit the bill when it comes to the particular phenomenal aspects of specific experiences. Particular phenomenal experiences occur in particular circumstances: we experience a sponge’s softness in the activity of squeezing it (Myin, 2003), “the stinging sharpness of a pin prick, the bitter-sweet taste of dark chocolate” (Schier, 2009) when we prick our fingers with a pin, or when we eat chocolate. The features of the interaction match the features of the experience. When we stop squeezing the sponge, or squeeze it too hard, the feeling of softness fades quickly, or gets replaced by feeling the hardness of one’s opposing hand. Pushing the pin in, accrues the pain, and brings it deeper into the body; the ways we handle the chocolate in our mouths, how we move it around, whether we bite or chew it, or let it slowly melt, affects, in predictable and controllable ways, the fine details of our gustatory experiences – just like putting glasses on drastically changes the visual experience of a myopic person in predictable and controllable ways (for many more examples of how interactive situation and in particular olfactory feel are related, see Cooke and Myin, 2011). In short, because person/environment interactions allow to meet both constraints dictated by general features of experience, and by the details of particular experiences, they seem to be appropriate terms to identify experiences with.

Of course, philosophers have argued, if not for the existence, then for the possibility of experiences divorced in some way from organisms, or from systems relevantly like organisms. Looked upon from the naturalistic perspective in which scientific explainability – broadly understood – stands central, arguments which only establish the possibility of disembodied experience, without any concern for explanation are not acceptable. That is, “dangling” possibilities, or possibilities which can be conceived, but which, if they would exist, would lack any explanation for why they would occur, are not acceptable for a naturalism which takes explainability serious. To come to this conclusion is to reiterate the prioritizing of explanatory concerns—avoiding “nomological danglers” (Feigl, 1958) – which has always been a prime motive for identity theorists. To illustrate: the idea of a momentaneous experience, that comes into and instantaneously goes out of existence because of a “quantum accident” (Clark, 2009)^2^, might in some modal sense be possible. Yet the fact that such occurrence would, by its very nature, be utterly unexplainable in even the most broadly construed naturalistic terms – it would be a miracle of sorts—renders it irrelevant as a consideration to draw conclusions from regarding the relation between minds, brains and bodies (elaborating on a point made in Myin, 2016: 100, see also Myin and Loughlin, 2018).

In contrast, the proposed identification of experiences and organismic environment-involving activities offers what seem exactly the right ingredients to explain conspicuous aspects of conscious experience such as perspectivalness or affect-ladenness. That is, these aspects of experience become more understandable after such an identification because the life of an organism provides an evaluative perspective from which organism-relative interests can flow forth, and from which subjective phenomena like desire, fear, pleasure and disgust can begin to be made sense of (Thompson, 2007, also Dennett, 1991, chapter 7). Moreover, biology gives us a platform to understand how such aspects gradually emerged, and evolved from “humble beginnings” into complex forms. Or, a biological, evolutionary framework provides us with the means to explain the coming into being of beings for which things, or matters, matter. Once there are organisms, with their (inter-)activity dependent ways of continued existence, the idea of a unique position from which to interact with that world, and the gradual development of forms of organization which benefit from the position created by the existence finds a foothold (Thompson, 2007; Kirchhoff and Froese, 2017)^3^.

The crucial move made by an embodied identity theory, so we propose, lies in the idea that telling the story of this gradual emergence of an organismic perspective, is the telling of the story of how experience, subjectivity and phenomenality emerged, and doing so in a gapless way. That is, during this evolution, it became something for the organism having such a perspective to have such perspective. But that “becoming” should not be cashed out in other terms than identity. What happened was not that some special ingredient became added to the mix, but rather that specific forms of organization came into existence. Being an organism having that form of organization, that is, actually occupying a particular perspective, living or enacting a life, is, for such an organism, to have experience.

Following the logic of identity exposed above, there is no further explanation to be given of why actually occupying such a perspective, being the organism that it is, coincides with being an experiencer.

Of course, there’s the recurring temptation to raise exactly this question for a further explanation. After all, being an organism and occupying a certain organismic perspective amount to objective facts and if they also hold the key to experience, an explanation is due of how objective facts can turn into subjective facts – or of how facts which can be adequately characterized by objective descriptions, can be identical to facts which fail to be fully captured in such objective descriptions.

This paradigmatic reasoning doesn’t respect the logic of identity, however. For if experience is actually identical to occupying an organism-bound perspective, it is not the case that objective facts turn into subjective facts. Rather, some objective events are identical to some subjective events.

Yet, crucially, one can relate in different ways to the facts of experience. One can enact, or live experiences or one can relate to them “from the outside”: by observing them or reflecting upon them. Simply said, and borrowing some vocabulary from Merleau-Ponty (1945), there’s “lived” experience and there’s “reflected upon” experience. For example, there’s actually engaging in certain perceptual interactions and there is reflecting on how one engages in certain perceptual interactions. The same events will be lived perceptual experiences in the one case, and related to as objective descriptions of a perceptual interaction in the other case.

The fact that taking an objective/descriptive stance toward something is itself an enacted experience, does not make it any more possible to occupy both the lived and the observational, reflective, or descriptive perspective on the same experience simultaneously. For it remains the case that a stance-taking experience is a different experience than the perceptual experience the stance is taken toward.

This impossibility is crucial, and provides a possible explanation for the tenacity of hard problems about phenomenal consciousness – a reason for why it seems to us that objective accounts of experience always contain gaps. Such seeming omissions might be explicated, not as the leaving out of something that more work from the objective perspective could provide, but rather as the very impossibility to take up both the subjective, experiential and the objective, reflective, and descriptive perspective at once. Attempting to take up these two perspectives simultaneously is like an attempt to see a Necker cube in both its spatial orientations at the very same moment. It simply cannot be done. However, our identity approach allows to recognize this impossibility problem for what it is. It is not the kind of problem on which we can expect a future science to deliver what is currently missing. We have not identified a gap in current scientific theorizing that, given enough patience, we can expect to be filled at some future point in time. Rather, the identity approach outlined allows us to recognize the problem as an impossible one to solve (Hutto and Myin, 2013, chapter 8. See also Zahnoun, 2018, chapter 5).

Again there might be a temptation to delve deeper. For other identities than those between the mental and the physical are different. For example, the perspective a Roman soldier had on water is different from the perspective a physical chemist has on H_2_O. This case is analogous to the Morning Star/Evening Star situation. Yet despite the differences, both perspectives are objective, reflective or descriptive. But in the case of the mind-body problem, one perspective is objective, and the other one not. Why this disanalogy? Our answer is that the root of the difference lies not in the existence of a queer new kind of objective fact, i.e., subjective facts, but because, in the case of organisms, but not in the case of water or planets, the subjective or experiential perspective exists – and that fact can be explained by invoking the biological history.

At this point, one could concur with our construal of the relation between the experiential and subjective on the one side and the objective, reflective and descriptive on the other and with the implication that the expectation of a scientific solution to the ‘Hard Problem of Consciousness’ is based on a mistaken view of the problem. Yet, so one could argue, this only raises a still deeper question, or a still harder problem, namely, the question why reality is such that experience arises at all – even if we have a satisfying diagnosis and therapy for our concerns. That is, perhaps one can accept that no further explanation is required for why some complex forms of organism-environment organization and interaction are identical to experience; and perhaps one can become convinced that the quest for a straightforward solution to hard problems of consciousness is not so much difficult as impossible. But, so one can insist, this still leaves the question of why reality is such that these forms of organism-environment interaction exist, and why reality is such that certain forms of organism-environment interaction are identical to experience with experiential qualities.

This is by all measures a reasonable concern. But notice that by understanding the mind/body problem in these terms, one is rephrasing it as an existential, rather than a scientific question. The questions asked now are questions about our place in the universe, as we find it, and as it remains even after everything is scientifically explained. Even more conspicuously so than it is the case for the original hard problem of phenomenality, this kind of question is one that should not, and cannot be solved in the way standard scientific questions are solved – irrespective of whether or not it can be solved in some other than scientific sense.

## Multiple Realization and Identity: What’s the Worry?

Our attempt to reinvigorate, or rather, reincarnate an identity proposal regarding the mind-body relation might be considered to be a pointless exercise. Weren’t identity proposals shipwrecked, once and for all, as soon as it was pointed out that, because specific types of mental states or processes can be realized by different types of physical structures, they can therefore not be identical with these physical structures? That is, doesn’t the fact that mental types (such as pain) can be multiply realized as tokens of different physical types (in a mammal’s brain, an octopus’s brain or an alien’s silicon brain), show that mental types are not identical to physical types? To this day, the argument from multiple realization (henceforth: MR) remains widely accepted, both as an argument against psychophysical identity, and against reductionist approaches to the psychological (see, for instance, Fodor, 1974, and for a critique, Bickle, 2003). However, the idea that the alleged multiple realizability of the mental can rule out possible psychophysical identities is not as solid as it prima facie might seem to be.

The thesis of the multiple realizability of the mental is defined as the claim that the same type of mental entity can be realized by different types of physical entities. We’re deliberately using the wide notion of ‘entity’ here so as to comprise the different kinds of things of which multiple realizability is being predicated in the literature. Multiple realizability is said to apply to states, processes, events or properties, depending on whose account one is considering. Moreover, accepting this thesis is supposed to entail a rejection of a possible mind-body identity theory.

First we should get clear on what the thesis of MR is supposed to be exactly. However, as Lawrence Shapiro points out, “despite philosophers’ ready acceptance of MRT^4^, it is not a precise thesis.” (Shapiro, 2000: 636) We think the imprecise nature of the MRT is the result of the imprecise nature of the elements that make up the thesis. On the one hand, multiple realizability is predicated of one and the same type. But what is a type, exactly? And what does it mean to attribute sameness to types? On the other hand, it is also unclear what the realization relation is supposed to be, exactly. Apparently, the relation has to be such that it rules out identity. But how, exactly, does it manage to do this? Also with regard to this question, we find no answer. Kim tells us in a footnote:

The term ‘realize’ used in [the multiple realizability principle] has not been explained. As we make progress... its meaning should become clearer; in the meantime, you will not go far astray if you read ‘P realizes M’ as ‘P is a neural substrate, or correlate, of M”’ (Kim, 1996: 102, fn. 4).

But how can neural entities be said to be a substrate of, or correlate with apparently abstract entities like types? In addition, it is also unclear when we are allowed to speak of a type being multiply realizable^5^. Lemons, oranges and grapefruits are all realizations of the type ‘citrus fruit.’ Does this mean that the type ‘citrus fruit’ is multiply realizable? But then, aren’t all types multiply realized in their token instantiations? But this would reduce the MRT to the utterly trivial ‘thesis’ that different things can belong in the same category. Considering these questions, it becomes clear that much of the MRT’s obscurity is the result of the fact that it is unclear to what extent the MRT requires a metaphysical commitment to an ontology of types, tokens, and a relation of realization between both. According to Thomas Polger^6^, the MRT should be given an empirical reading which avoids the nagging metaphysical issues above. Polger claims that the MRT “is most plausibly thought of as the claim that psychological state kinds are shared in common across at least some physical creature kinds, for example, across species.” (Polger, 2013: 870) Furthermore, this is said to come closest to what Hilary Putnam had in mind when he first presented MR as an argument against mind-brain identity. Recall this oft-quoted passage:

Consider what the brain-state theorist has to do to make good his claims. He has to specify a physical-chemical state such that any organism (not just a mammal) is in pain if and only if (a) it possesses a brain of a suitable physical-chemical structure; and (b) its brain is in that physical-chemical state. This means that the physical-chemical state in question must be a possible state of a mammalian brain, a reptilian brain, a mollusc’s brain (octopuses are mollusca, and certainly feel pain), etc (Putnam, 1975: 436).

Putnam proposes it is extremely unlikely that the identity theorist will be able to “make good his claims” because of the fact that creatures with different brains can nevertheless be in the same mental state (pain). His claim is clearly supposed to be empirical in nature:

I shall not apologize for advancing an empirical hypothesis. Indeed, my strategy will be to argue that pain is not a brain state, not on *a priori* grounds, but on the grounds that another hypothesis is more plausible. (Putnam, 1975: 433).

With these manoeuvers, Putnam has reframed the identity theory as hinging on a claim about the relation between tokens of mental phenomena and types. Apart from the already indicated unclarities about core concepts crucially involved in the steps in which this reframing takes place, the whole enterprise rests, so we will now argue, on a deeper confusion about what the identity theory is about. In a nutshell, as we construe it, the core claim of an identity theory is one about relating, on the one side the experiential, as encountered from an experiential perspective, and about what’s encountered from an onlooking, objective, reflective or descriptive perspective. The crucial claim of an identity theory consists in spelling out that, despite initial appearances, the experience – the experience itself, not its description – is not different from what can also be referred to in an objective way, via observations, and in particular via descriptions. In other words, the identity theory wants to make a connection between, on the one hand, the realm of experience, characterized by subjectivity, and the realm of the objective, characterized by observation, reflection and description, on the other hand. Putnam’s reframing of the identity theory, in contrast, construes the theory as aiming to make connections within the realm of the objective, reflective and descriptive, in particular between descriptions of physical events in particular or general terms and descriptions of mental events in particular or general terms. But this is a completely different project. The difference between objective versus subjective ways or modes of encountering is a different difference than the difference between descriptions of the physical and descriptions of the mental, irrespective whether these descriptions are particular or general (token or type). The thesis that two pains can be characterized with the same mental description, yet not with the same physical description, is a thesis about how different descriptions of the experiential relate, not about how the experiential relates to the descriptive. As a consequence, Putnam’s considerations don’t even touch on what the identity theory, as we have construed it, is about.

In other words, the MR challenge is simply misplaced for the identity theory (as we take it) as a philosophical view on experience. It is, literally, besides the question.

Our rejection of the relevance of the argument from MR for the identity theory pivots on the difference between experience and its description. Embodied views of experience as enacted organism-environment interaction allow to bring home the point forcefully. For such embodied views allow to see experience purely as organism-environment interaction (Hutto and Myin, 2013, 2017; Raleigh, 2015). In particular, such enacted experience needn’t have any descriptive representational content. It is specific to certain circumstances, and to a certain organism, and it can perhaps be less or more appropriate to certain circumstances. Yet despite such specificity, the experience does not specify, or carry a semantically evaluable content, which is about those circumstances which it is a specific reaction to – or embedded interaction with. Not carrying descriptive content, it doesn’t form either a particular or a general description: the experience of pain doesn’t self-describe as a token brain state, a token pain, a type pain, or a type brain state. On such understanding of experience, the tension between the experiential and the descriptive is a natural fact.

Of course, one can make explicit in one’s description of an experience that one is focusing on a particular experience, or that one is talking about the similarities between experiences. Here, at the level of descriptions, the difference between tokens and types makes sense. And then one can ask questions about the relations between descriptions, for example whether a second more general (type) description applies, or does not apply to two tokens to which a first more general description applies. But these are questions regarding the relations between descriptions, and not relations between a phenomenon and descriptions. The core identity claim is about the latter: about the phenomenon of experience and the realm of objective descriptions. What’s asserted is that, whenever we identify something subjectively as a mental entity (a toothache, say), we can in principle also descriptively identify it as some physical entity, because it is just one thing, but related to in different ways (subjectively experienced vs. intersubjectively described)

Of course, it matters which descriptions are used at the objective/descriptive side, when making the connection between the experiential and the objective. What’s on the objective side should be characterized in terms of organismic doings, objectively described. That is, if our embodied identity theory is right, it should be a description which picks out the naturalistically intelligible conditions in which consciousness actually occurs. Naturalistically intelligible here means: explainable, as we have indicated. That disqualifies any form of functionalism that holds that there is something like a functional(ist) level, on which the functional kinds reside. What we object to is any functionalism that makes the *a priori* claim that the mental should be characterized in terms of multiply realizable functional types.

The question is: how can one know, *a priori*, that such a functional level of multiply realizable types exists that are optimal for the description of mentality? Recall Polger, who rightly states that, on an empirical, naturalistically acceptable understanding of functional types, “psychological state kinds are shared in common by at least some physical creature kinds, for example, across species.” (Polger, 2013: 870) This formulation doesn’t speak of an unexplicated realization relation, yet it does contain the idea that one and the same mental type (or kind) can be shared by creatures of a different physical kind. Leaving aside the important questions of what is to count as sufficiently similar or different and what not, note that Polger’s reformulation still speaks of types or kinds, which are now said to be shared. Strictly speaking, however, the idea that one and the same type can actually be shared by different creatures is, when taken at face value, again the expression of a specific metaphysical assumption, namely the assumption that the occurrence of a certain mental event needs to be ontologically understood in relation to types. On this account, saying that two different creatures can both have pain needs to be understood in terms of these two creatures sharing a type. But again, from an empirical, naturalistic, point of view, how can an abstract entity like a type literally be “shared” or “distributed” amongst different creatures? To reformulate the idea that the mental should be described in terms of multiply realizable functional types as a fully fledged empirical hypothesis, we also need to cash out the notion of ‘type’ in empirically respectable terms. It would be a reification of ‘types’ to think of them as individual things with their own, perhaps non-spatiotemporal existence, things that can moreover be distributed amongst other things, or that can manifest themselves in physical incarnations. Rather, from an empirical, naturalistic point of view, types are best understood as the names of the accepted categories in accordance to which we, in a certain community, structure the world, in the light of this or that purpose. In other words, saying that the mental type ‘pain’ is shared by a human being, a cat and an octopus is simply a way of saying that these animals can sometimes have a sensation which we assume to be relevantly similar (i.e., according to an accepted classificatory criterion) so as to allow the identification of both sensations as pains. Simply put, on the empirical reading, claims cast in terms of shared functional types state that creatures which we classify as relevantly different in physical respects (according to some accepted criterion) can be in a mental state, or have a kind of experience, which we classify as relevantly similar (again, according to some accepted criterion). Reformulating Putnam’s example, he apparently held an octopus brain to be relevantly different from human brains, yet he claimed that octopi and humans can nevertheless have experiences which are relevantly similar enough so as to warrant the label ‘pain.’

The crucial point to be made here is that whether the relevant similarities obtain is not something which can be decided in any *a priori*, decontextualized way. Importantly, which similarities are relevant depends on the context. In some contexts, differences which might matter in other contexts, might be disregarded. For example, one might talk about a general class of analgesic substances, but in some contexts (such as avoiding allergic reactions), very specific chemical details might matter. These are well known problems having to do with the grain of functional analysis: there is no one level of properly ‘functional’ causality below which anything else is ‘implementation detail,’ or any dichotomous division of natural properties into the ‘functional’ versus the ‘structural’. Most importantly, whether or not functional descriptions, at some level, are multiply realizable, in some context, seems to be something that can only be empirically established, by investigating the cases, and finding out about how similar or dissimilar these different ‘realizations’ behave. Of course, all of this already presupposes that we agree on criteria by which to judge what is, and what isn’t relevantly similar or dissimilar.

We are not merely reiterating the points made by Putnam regarding functionalism as “advancing an empirical thesis.” On the contrary, although we agree that specific functionalist claims to the extent that this or that functional kind is multiply realizable are empirical, we are emphasizing that assessing the plausibility of such hypothesis should proceed in an empirical way: by considering actual cases, instead of by *a priori* argument irrespective of such cases^7^. This raises doubt on one immensely influential line of argument in favor of functionalism, and against the identity theory. We have in mind arguments that flow forth from the fusion of functionalism with widely accepted ideas about information processing. In a nutshell, it is widely accepted that information, and by implication information processing, is something that is independent of the material medium in which the informational processes take place. This seems obviously so when the information at issue is semantic. The same semantics can be carried by something printed on paper, grafted in stone or transferred by acoustic waves. But exactly the same seems to hold for other kinds of information, such as information based on covariation, or information understood to be processes in or by computing machines. Irrespective of its physical makeup, anything that has the right causal connections with something else can be said to carry information about that second thing in a co-variational sense. And it is a well-known engineering fact that computers can be made, and be made to carry out exactly the same computations, by a wide variety of material means. Now, if one also holds that cognition is some form of information processing, and if it hardly requires argument that information processing is independent of physical substrates, cognition itself becomes evidently, without requiring further investigation or consideration of cases, matter or medium independent, or multiply realizable (for such reasoning, see for example Piccinini, 2015).

However, as many have argued, there are good reasons to take cognition to be embodied interaction instead of information processing (see, for instance, Hutto and Myin, 2013, 2017). If cognition is embodied interaction, instead of information processing, though prima facie it remains a possibility that cognition is multiply realizable in some non-trivial and scientifically interesting sense, this doesn’t follow with the apparent immediacy it does within an information processing framework.

Suppose, for example, that non-representational, non-information processing dynamical systems accounts of cognition are correct, according to which cognition is organism-environment adaptation or coordination at multiple temporal and spatial scales at once. During such multi-scale interactions, structures dynamically emerge and stabilize, but on such an account, these are physical changes within the coupled organism-environment system rather than the acquisition of representations or the processing of information. Due to the occurrence of the multi-scale changes, organisms become able to deal with the current environment in a way which is sensitive, adapted or attuned, to what is currently strictly speaking absent, or abstract. If embodied and embedded cognitive systems are dynamical systems in the way sketched here, this has important implications for multiple realizability. Cognitive phenomena then are phenomena which occur under specific conditions of massive complexity. As they might require multiple levels of interrelated and mutually sustaining structure and coordination, they might only be possible in certain kinds of systems. Some philosophers, sympathetic to a dynamical/ecological perspective (Di Paolo and Thompson, 2014), have argued that the kind of structural dynamics required, is the self-sustaining, self-creating dynamics of living systems. Further, the interdependence characterizing such systems allows to recognize the role of bodily processes and structures as fundamental to cognition. If the cognitive activities of an organism as a whole depend on the ways in which its parts are coordinated on multiple scales, what one does with one’s hands while speaking becomes an integral part of the process of speaking, a thesis that is congruent with recent theorizing on gesture (see Goldin-Meadow and Alibali, 2013). And what goes for the body, goes for the environment, in a broad sense, so that it includes not only one’s physical, but also one’s sociocultural context (see Spivey and Spevack, 2017) for a maximally inclusive account of cognition).

We think that the prospects for the multiple realizability of cognition would vastly change if such a dynamical model of cognition held true, instead of a computational, or information processing model. For while it would remain possible to model such a system mathematically^8^, this would not mean that what’s modeled would actually be multiply realizable – in contradistinction to being ‘simulatable’ – in materially different substrates. It could not be easily dismissed that only one thing can actually have that dynamical structure belonging to the whole complex system. Of course, one could only simulate that structure, but that would be a simulation, not an actual “instantiation” – not something that had that structure. We leave it to further work to pursue the issue of the relation between embodied cognition and multiple realizability of the structure. But what has been said already sufficiently underscores the general point we want to make here: that multiple realizability should, apart from being contextualized, not only be taken as an empirical thesis, but that one should also not too rapidly conclude, on the basis of very general considerations, that it actually applies. Attention to the actual specifics of cognition, highlighted by embodied approaches to cognition, can help to counteract that tendency. We take this to form a welcome side-benefit of (re-)incarnating the identity theory.

## Identity and Reduction

If our embodied identity theory is not defeated by multiple realization arguments, it should be clear that it also is not vulnerable to another famous line against it, due to Searle, namely that it disregards the mind. Such complaint might apply to versions of the identity theory in which identity is lumped together with reductionism, or at least, ontological reductionism. These reductionist interpretations are typically expressed through, what Donald Davidson calls, the “nothing-but” reflex (Davidson, 1980: 214). The claim that the mental is identical with the physical is almost always understood as synonymous with the ontologically reductive materialist claim that the mental is ‘nothing but’ the physical, or that, more specifically, humans are ‘nothing but’ physico-chemical mechanisms. It follows, then, that if one takes identity theory to require a commitment to ontologically reductionist materialism, any argument against this kind of reductionism is by default an argument against identity theory. Indeed, we do find reductionist tendencies within the classic identity proposals. Consider, for instance, these lines from Smart.

It seems to me that science is increasingly giving us a viewpoint whereby organisms are able to be seen as physicochemical mechanisms… That everything should be explicable in terms of physics (together of course with descriptions of the ways in which the parts are put together-roughly, as biology is to physics as radio-engineering is to electromagnetism) except the occurrence of sensations seems to me to be frankly unbelievable (Smart, 1959: 142).

And in later work, we read:

I shall be concerned to put man in his place by defending the view that he is nothing more than a complicated physical mechanism.… I wish to argue for the view that conscious experiences are simply brain processes (Smart, 1963: 15 & 88, m.e.).

The reductionist nature of these assertions is undeniable. And also in Place’s classic paper, we find support for a reductionist interpretation of identity when he writes that, to all identity statements (statements using the ‘is’ of composition), we can add the qualification ‘and nothing else’, so that we could say that consciousness is a brain process, and nothing else (see Place, 1956: 45). To the extent that these accounts should be read as ontologically reductionist, they are eliminativist with regard to the phenomenal. Indeed, some have identified this alleged eliminativism as the identity theory’s greatest weakness (next to its inability to deal with the multiple realizability of the mental). In his 1992 The Rediscovery of the Mind, Searle puts the objection as succinctly as possible when he claims that identity theory “leaves out the mind.” (Searle, 1992: 53) But if an identity theory can really be said to be at bottom an eliminativist materialism, we should start to wonder whether this theory can still be probably labeled an identity theory. The problem, after all, is that a relation of strict identity can never be a relation of ontological reducibility, for the simple reason that identity is symmetrical, whereas the ontological reductive relation is not. If A is strictly identical with B, then, of course, B is also strictly identical with A. But saying that B reduces to A, on the other hand, does obviously not entail that A reduces to B. So if conscious experiences are really strictly identical with brain processes, as the classic identity theorist claims, we might just as well hold that these brain processes are ‘nothing but’ conscious experiences, or that certain physico-chemical mechanism are simply humans, and nothing else. For as Davidson aptly points out: “[I]f some mental events are physical events, this makes them no more physical than mental. Identity is a symmetrical relation” (Davidson, 1987: 453)^9^.

In any case, whether the eliminativist reading of some of the classic versions of the identity theories of Smart, Place and Armstrong is apt, Searle’s complaint that the theory leaves out the mind does certainly not apply to the version of the identity theory we have defended here. For our version, rather than denying, recognizes and affirms the specificity of the enacted perspective of experience. Yet it warns not to take this perspective for something it is not. That is, the perspective should be taken for a distinctive way of encountering experience, and not as a way of encountering something distinctive from the rest of nature.

## Conclusion: The King is Dead, Long Live the King

The identity theory, despite having been officially declared dead, still has a future. Its vital core, the logic of strict identity, allows to deal with vexed questions about the relation between experience and objective facts. Yet while the identity theory must retain this logic at its heart, it needn’t necessarily remain a mind/brain identity theory. Allying with embodied ways of thinking about the mind, it can become the embodied identity theory. Such embodied turn allows for a naturalistically, evolutionary account of perspectivalness, and it enables to forcefully motivate the rejection of the idea that multiple realizability undermines identity approaches. Instead of disregarding experience, the embodied identity theory gives it a central place. Chapter 3 is up for considerable revision.

## Author Contributions

Both authors were involved in the conception, elaboration and refinement of the ideas, and arguments in this paper. The idea of an embodied identity theory, and it being concerned with the relation between experience and descriptions of experience are mainly due to EM. FZ helped to refine and streamline these ideas, and added material in particular on multiple realization, as well as on the historical and dialectical context of the identity theory. Further, he devised the section on reductionism.

## Conflict of Interest Statement

The authors declare that the research was conducted in the absence of any commercial or financial relationships that could be construed as a potential conflict of interest.

## Footnotes

1 The point has also been made on several occasions by Papineau (1998, 2002). See, for instance, Papineau (1998: 373, 2002: 12).

2 Note that this is a misnomer, the intricacies of quantum mechanics are not nomological danglers in this sense.

3 This leaves open what exactly an organism is, and which organisms have experiences. We think it is not the task of an identity theory to decide these issues. It is in biology, in a broad sense which includes philosophy of biology where it is discussed what counts as an organism. Similarly, the specification of what “experience” is, is an interesting and crucial issue, but there is no reason why one should expect that an identity theory should offer the means to decide where exactly experience begins – if, in the light of the gradualness of evolution such boundaries exist at all. There is a division of labor concerning these matters. A court case offers an analogy. Whether some act qualifies as a crime, and what the facts are in a certain case, is determined by lawmaking, and by police or detective investigation. The task of a lawyer in court begins when these tasks have already been finished. The lawyer’s task will be to motivate, against the established legal and factual background, why a certain identification should, or should not be made. Note that Polger (2004, chapter 2) defends an identity theory, while holding that we currently have no criteria for counting something as an experience.

4 Multiple Realizability Thesis.

5 Shapiro seems to be mainly concerned with the question of what is, and what isn’t supposed to count as an instance of multiple realization. We, however, shall be focusing more on the other issues, as these are directly relevant for our discussion about MRT’s relation to identity theory.

6 See Polger, 2013.

7 This point is also repeatedly emphasized in the work of Shapiro, Polger and Polger and Shapiro. See, for instance, Shapiro (2000), Polger (2004, 2009), and Polger and Shapiro (2016).

8 All mathematical models are abstract, and thus in principle multiply realizable. But this doesn’t imply they are actually multiply realized.

9 In this regard, it should be stressed that Davidson’s anomalous monism is not to be understood as a form of physicalism or materialism, be it reductive or non-reductive. Davidson is very explicit about this: “Anomalous Monism is not a form of physicalism or materialism” [(Davidson, 1995): 75].
